# Can local infiltration analgesia supplemented with tranexamic acid reduce blood loss during total knee arthroplasty?

**DOI:** 10.1186/s12891-024-07451-9

**Published:** 2024-04-26

**Authors:** Łukasz Wiktor, Bartłomiej Osadnik, Maria Damps

**Affiliations:** 1 Department of Trauma and Orthopaedic Surgery, Upper Silesian Children’s Health Centre, Katowice, Poland; 2 Department of Trauma and Orthopedic Surgery ZSM Hospital, Pokoju street 74, Chorzów, 41-500 Poland; 3 Department of Anaesthesiology and Intensive Care, Upper Silesian Children’s Health Centre, Katowice, Poland

**Keywords:** Knee, Arthroplasty, Tranexamic acid, Local infiltration analgesia, Blood loss

## Abstract

**Purpose:**

The aim of this study was to investigate the efficacy of TXA supplemented with local infiltration analgesia (LIA) for reducing blood loss in patients undergoing total knee replacement.

**Materials:**

A retrospective study of 530 individuals with a mean age of 71.44 years was performed after posterior stabilized total knee arthroplasty. Patients were divided into three groups according to the method of bleeding control: I - patients without an additional bleeding protocol (control group); II - patients receiving IV TXA (TXA group); and III - patients receiving the exact TXA protocol plus intraoperative local infiltration analgesia (TXA + LIA group). Blood loss was measured according to the maximal decrease in Hb compared to the preoperative Hb level.

**Results:**

The mean hospitalization duration was 7.02 (SD 1.34) days in the control group, 6.08 (SD 1.06) days in the TXA group, and 5.56 (SD 0.79) in the TXA + LIA group. The most significant decrease in haemoglobin was found in the control group, which was an average of 30.08%. The average decrease in haemoglobin was 25.17% (*p* < 0.001) in the TXA group and 23.67% (*p* < 0.001) in the TXA + LIA group. A decrease in the rate of allogeneic blood transfusions was observed: 24.4% in the control group, 9.9% in the TXA group, and 8% in the TXA + LIA group (*p* < 0.01).

**Conclusions:**

Compared to the separate administration of tranexamic acid, the combination of perioperative administration with local infiltration analgesia significantly reduced blood loss in patients after total knee replacement.

**Supplementary Information:**

The online version contains supplementary material available at 10.1186/s12891-024-07451-9.

## Introduction

Osteoarthritis is one of the most common reasons for disability. Furthermore, treatment after such a diagnosis requires significant financial resources. Total knee arthroplasty (TKA), next to total hip arthroplasty (THA), is one of the most commonly performed elective orthopaedic surgeries. The main tasks are to reduce pain, restore knee function, and improve quality of life. Although knee replacement is an effective treatment for severe joint damage, postoperative complications, including blood loss, thrombosis, infection, and loosening or malalignment of the prosthetic component, are known. Due to the blood loss associated with TKA, which can range from 1500 to 2000 mL, the extent of perioperative bleeding has been debated for many years [1].

Blood loss can be evaluated either by an estimation of the loss or by measurements of the drop in haemoglobin (Hb) and haematocrit (HTC) as a surrogate for blood loss [2, 3]. Different blood-sparing techniques for TKA, such as hypotensive anaesthesia, perioperative blood salvage, perioperative blood donation, and recombinant human erythropoietin, have been studied. Every 1000 mL of blood loss decreases the haemoglobin concentration by approximately 3 g/dL [4]. Significant blood loss increases the risk of blood transfusion and may result in prolonged hospital stay and delayed rehabilitation. Postoperative allogeneic blood transfusion is a considerable risk factor for haemolytic and nonhemolytic reactions. Moreover, the costs related to knee replacement increase [5, 6]. Enhancing the surgery with a tourniquet promotes activation of the fibrinolytic system, and hyperfibrinolysis is considered the primary cause of postoperative bleeding after TKA surgery [7]. Tranexamic acid (TXA) is a fibrinolysis inhibitor that directly prevents plasminogen activation and inhibits activated plasmin from degrading fibrin clots. TXA promotes haemostasis and can reduce the duration of bleeding and the quantity of blood loss [8, 9]. Notably, TXA has been included in the List of Essential Medicines of the World Health Organization (WHO) and is currently utilized in many different fields of medicine [10]. Clinical trials, systematic reviews, and meta-analyses have confirmed that intravenous and topical TXA administration effectively reduces blood loss and transfusion risk. Intravenous infusion of TXA is the most common method, but many studies have shown that topical application of TXA has similar safety and efficacy in TKA patients [11, 12]. The analgesic effect associated with intraoperative topical use of TXA seems interesting and is most likely associated with decreased postoperative inflammation and surgical site swelling [11, 13]. Several perioperative TXA management protocols, including a single dose and multiple administrations, have been discussed [14–16]. Recently, additional studies have focused on the administration of oral TXA [17, 18].

### Local infiltration analgesia (LIA)

A technique including high-volume local infiltration analgesia (LIA) developed by Kerr and Kohan was used for analgesia after TKA and THA [19]. The initial assumption was to infuse ropivacaine, ketorolac, or adrenaline into the tissues around the surgical field and supplement with additional postoperative intra-articular injections. Despite limited data, nonsteroidal anti-inflammatory drug (NSAID) use is associated with improved management of postsurgical pain [19]. Currently, LIA has been used for pain relief for more than 15 years in patients who have undergone total knee replacement.

We investigated the efficacy of TXA supplemented with LIA for reducing blood loss in patients undergoing TKA.

## Materials and methods

The STROBE protocol was designed for retrospective observational studies [20].

Patients diagnosed with osteoarthritis who underwent unilateral primary cemented TKA at ZSM Hospital in Chorzów between January 2018 and July 2022 were enrolled in the study. The following inclusion criteria were established for further evaluation to assess homogeneity between the groups: (1) patients who underwent posterior-stabilized (PS) TKA exclusively because this procedure requires the release of the posterior joint capsule and is at risk of more significant intraoperative blood loss; (2) patients who underwent spinal anaesthesia (the vast majority of patients) because general anaesthesia may result in increased blood loss; and (3) patients who underwent surgery by one of the two surgeons (Ł. W; B.O.). Patients with (1) preexisting coagulopathy; (2) a history of long-term anticoagulant therapy before surgery; (3) a history of other additional procedures, including implant removal; and (4) any complications requiring extended surgery, e.g., intraoperative femoral condyle fracture, were excluded from the evaluation. After considering the above criteria, 530 individuals were included for further analysis. All the data have been anonymized. The authors had no access to information that could identify individual participants. Patients were divided into three groups corresponding to the method of bleeding control: I - patients without an additional bleeding protocol (control group); II - patients receiving 1,0 g of TXA administered intravenously 30 min before the skin was incised and 3 h later (TXA group); and III - patients receiving an exact TXA protocol combined with intraoperative local infiltration analgesia (200 mg of ropivacaine and 0.5 mg of epinephrine in 100 ml of solution) (TXA + LIA group). The difference in the number of individuals between groups resulted from restrictions related to COVID-19 during the study period.

Based on the Polish guidelines for preventing and treating venous thromboembolism, each patient received a dose of low-molecular-weight heparin (LMWH) 12 h before surgery (40 mg Clexane, Sanofi, Paris, France), and LMWH was continued postoperatively for 14 days. To assess blood loss, the haemoglobin (Hb-g/dl) level before surgery was compared with the lowest haemoglobin level during the hospital stay. As a standard, laboratory tests were performed on the day of hospital admission, the second postoperative day, and the day preceding discharge. The rate of blood transfusions was assessed in each group. According to local hospital regulations, the haemoglobin **cut-off** level for transfusion was 8.5 g/dl. However, for patients with anaemia-related symptoms, the threshold value indicating the need for transfusion was less than 9.0 g/dl.

The patients were followed up until one month after discharge to assess the early readmission rate.

### Surgical technique

The two most experienced knee arthroplasty surgeons performed surgical procedures (Ł. W and B. O, each performing more than 150 posterior stabilized knee arthroplasties annually). Only patients who underwent surgery under spinal anaesthesia were analysed. All patients in the study groups were anaesthetized by the same group of anaesthetists dedicated to the orthopaedic theatre. In accordance with the anaesthesia protocol for knee arthroplasty in our centre, the patients’ mean systolic blood pressure during surgery was controlled and maintained in the 100–110 mmHg range.

**Fig. 1:**
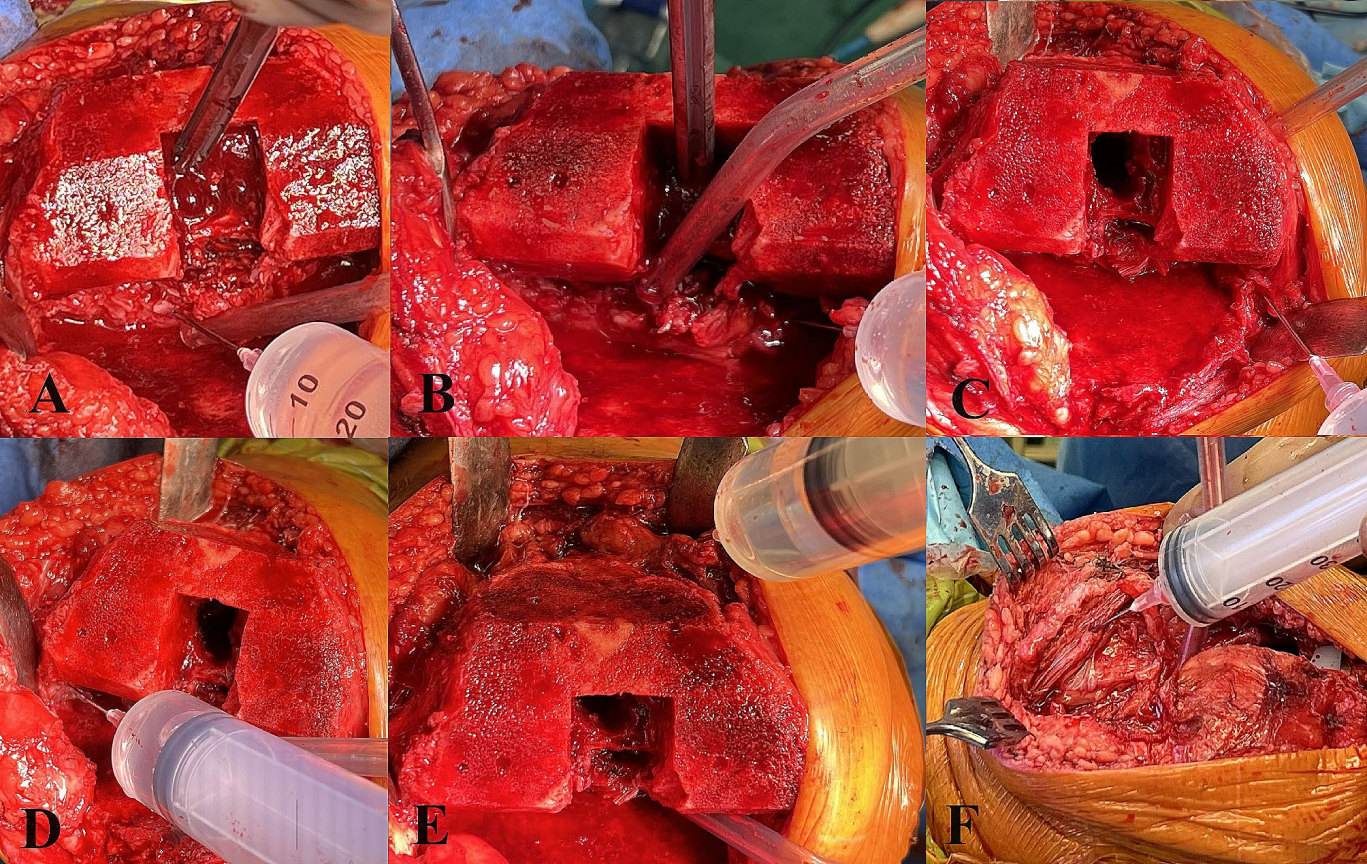
Intraoperative images showing LIA administration: posterolateral **(A)** and posteromedial **(B)** joint capsule, medial collateral ligament **(C**), lateral collateral ligament **(D)**, supracondylar soft tissue **(E)**, and quadriceps tendon **(F)**

All patients underwent a standard medial parapatellar approach through a straight midline incision of the quadriceps tendon. Surgical procedures were performed without a tourniquet, and sufficient haemostasis was achieved. The drain was used only in the control group and was obligatorily removed 24 h after the procedure, nonetheless because of the drainage amount. When TXA was included in the perioperative protocol, drains were not used. After standard bone cuts, reasonable efforts were taken to obtain a correct soft tissue balance to keep the joint aligned during flexion and extension. The bone surfaces of the patients receiving cement were cleaned by using a pulsed lavage system to remove fat residue, bone debris, marrow, and blood. The LIA solution contained 200 mg of ropivacaine and 0.5 mg of epinephrine in a volume of 100 ml. The solution was sterile and prepared in two 50 ml syringes. LIA was performed in two stages. The first dose (30 ml) was injected into the posterior joint capsule directly after osteophyte resection on the posterior aspect of the femoral condyles, followed by the release of the tightened capsule (Fig. 1A and B). The midpoint of the posterior capsule was avoided due to the proximity of the neurovascular bundle. The second dose was injected into the remaining parts of the joint: the medial collateral ligament (10 ml; Fig. 1C), lateral collateral ligament (10 ml; Fig. 1D), supracondylar soft tissue (20 ml; Fig. 1E), quadriceps tendon (10 ml; Fig. 1F), and subcutaneous tissue (20 ml). Each time, we ensured that LIA was administered in accordance with the accepted standard, using the same step-by-step procedure for each patient.

For all patients, the joint capsule was tightly sutured using STRATAFIX Symmetric PDS Plus 1 − 0 (ETHICON, Johnson & Johnson, Germany). After the surgery, a compression dressing was applied on the operated limb. On the first postoperative day, weight-bearing was allowed in accordance with a standard rehabilitation program.

The study was conducted according to the guidelines of the Declaration of Helsinki. For the study, ethical approval and participant consent were waived by the bioethics committee of the Silesian Medical Chamber in Katowice, Poland (ŚIL.KB.1011.2022) due to the retrospective nature of the study.

All the data were fully anonymized before analysis.

## Results

Patients were divided into groups according to the inclusion and exclusion criteria corresponding to the protocol for reducing blood loss and the need for a blood transfusion. The TXA + LIA group had the most patients, with 225 patients. There were fewer patients in the control group and TXA group, 164 and 141 patients, respectively. All the groups were homogeneous in terms of sex, with a female predominance (74.4% in the control group, 70.2% in the TXA group and 75.5% in the TXA + LIA group). The mean age of the patients was 71.44 years. Only the age of patients in the TXA group requiring blood transfusion differed from this value, with a mean age of 75,93 years. The mean BMI for all patients was 27.47 kg/m2. None of the 20 patients in the control group (12.2%), 19 patients in the TXA group (13.5%), and 31 patients in the TXA + LIA group (13.7%) had comorbidities. The physical status of the patients was also analysed according to the American Society of Anaesthesiologists (ASA) classification. We did not observe any significant differences between the individual study groups. The ASA classifications of each group are as follows: control group ASA I – 11, ASA II – 107, ASA III – 46; TXA group ASA I – 9, ASA II – 94, ASA III – 38; and TXA + LIA group ASA I – 14, ASA II – 149, ASA III – 62.

**Fig. 2:**
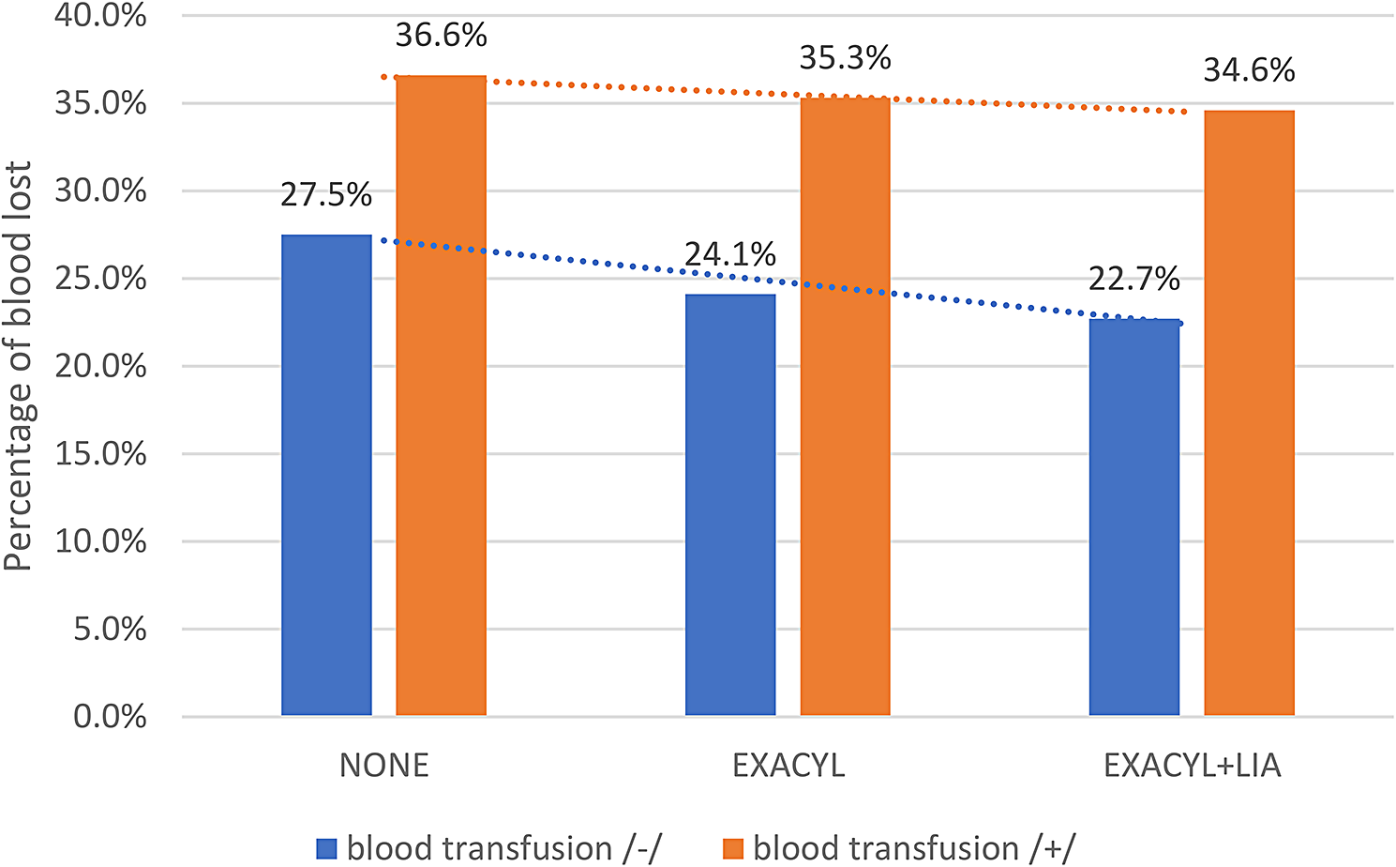
A bar chart showing the percentage of blood loss relative to the preoperative value for all study groups of patients requiring (blue) and not requiring (orange) blood transfusion

The mean hospitalization duration was 7.02 (SD 1.34) days in the control group, 6.08 (SD 1.06) days in the TXA group, and 5.56 (SD 0.79) in the TXA + LIA group. An overview of the study group is presented in Table 1. The most significant reduction in haemoglobin was found in the control group, which was an average of 30.08%. The average decrease in haemoglobin was 25.17% (*p* < 0.001) in the TXA group and 23.67% (*p* < 0.001) in the TXA + LIA group. The difference between the TXA and TXA + LIA groups was statistically significant, with a mean difference of 0.05 (-0.28757; *p* = 0.02). Perioperative TXA administration significantly reduced blood loss (*p* < 0.01), as indicated by a 3.4% decrease in haemoglobin compared to the preop haemoglobin value and a reduction of 4.8% in TXA + LIA patients not requiring a transfusion (*P* < 0.01). Perioperative TXA administration significantly reduced blood loss by 1.3%, and TXA + LIA did so by 2% for patients requiring blood transfusion. Figure 2 shows the percentage of blood loss compared to the initial haemoglobin value (separated by groups of patients who required and did not require blood transfusion). Based on the interdependence statistics (chi-square test = 23.894; *p* < 0.01), there was a statistically significant relationship between the decrease in the rate of allogeneic blood transfusions, which was 24.4% in the control group, 9.9% in the TXA group and 8% in the TXA + LIA group.

**Table 1:** Overview of the study group

	No protocol group		TXA group		TXA + LIA group	
	blood transfusion /+/	blood transfusion /-/	blood transfusion /+/	blood transfusion /-/	blood transfusion /+/	blood transfusion /-/
No	40	124	14	127	18	207
Age [mean]	70,82	68,98	75,93	69,77	72,61	70,57
Sex [M/F]	8 / 32	34 / 90	2 / 12	40 / 87	2 / 16	53 / 154
BMI	27,625 (SD 1,93)	27,04 (SD 1,99)	27,80 (SD 1,98)	26,97 (SD 2,03)	27,96 (SD 1,59)	27,46 (SD 2,3)
Hospital stay [day]	7,32 (SD 1,55)	6,92 (SD 1,26)	6,71 (SD 0,82)	6,01 (SD 1,06)	5,88 (SD 0,9)	5,54 (SD 0,78)
Preoperative Hb (g/dl)	13,36 (SD 0,95)	14,13 (SD 1,15)	12,83 (SD 0,74)	14,13 (SD 1,18)	12,37 (SD 1,19)	13,81 (SD 1,15)
Lowest Hb (g/dl)	8,25 (SD 0,51)	10,24 (SD 1,09)	8,3 (SD 0,36)	10,71 (SD 1,14)	8,06 (SD 0,69)	10,67 (SD 1,21)
Maximum Hb loss (g/dl)	5,11 (SD 0,90)	3,89 (SD 0,82)	4,53 (SD 0,50)	3,41 (SD 0,98)	4,31 (SD 0,92)	3,14 (SD 0,90)

### Complications

In seven patients (5 in the control group and 2 in the TXA group), we observed persistent wound leakage lasting more than 72 h, requiring prolonged hospitalization and proper wound care. One patient from the control group was readmitted with symptoms of wound infection four weeks after the initial surgery. The patient underwent irrigation and debridement (I & D) with polyethylene insert replacement, and the prosthesis was retained. No pathogens were cultured from the intraoperatively collected materials. After I & D, no symptoms of infection recurred. We documented two cases of deep venous thrombosis among 530 patients (one in the control group and one in the TXA + LIA group).

### Statistical analysis

Two-factor ANOVA was used for analysis of the test results. This analysis verified whether the study groups differed significantly in terms of blood loss. This work used a multivariate analysis technique utilizing CHAID prediction trees with independent component analysis. BMI, age, and sex were assumed to be independent secondary variables. Pearson’s correlation coefficients were calculated to assess the associations between independent secondary variables and blood loss. Tukey’s post hoc test for unequal samples was subsequently used to analyse the detailed results. Before starting the analysis of differences, descriptive statistics were calculated (Table 2).

**Table 2:** Descriptive statistics. M - mean; SD - standard deviation; MD – median; MIN - minimum; MAX – maximum

		blood transfusion /-/				blood transfusion /+/			
		hemoglobin (Hb-g/dl)		decrease	%	hemoglobin (Hb-g/dl)		decrease	%
		post-op	pre-op		decrease	post-op	pre-op		decrease
Control group	M	10,2	14,1	3,9	27,5%	8,3	13,4	5,1	38,0%
	SD	1,1	1,2	0,8	5,2%	0,5	1	0,9	4,9%
	MD	10,1	14	3,8	26,6%	8,5	13,5	5,1	37,0%
	MIN	8,3	11,9	1,9	13,7%	7	10,3	2	19,4%
	MAX	15,2	19	6,5	40,9%	8,9	15,4	7,3	49,3%
	N	124				40			
TXA	M	10,7	14,1	3,4	24,1%	8,3	12,8	4,5	35,3%
	SD	1,1	1,2	1	6,3%	0,4	0,7	0,5	2,2%
	MD	10,7	14	3,5	24,6%	8,4	12,7	4,5	35,4%
	MIN	8,8	10,9	0,4	3,4%	7,5	11,8	3,8	32,2%
	MAX	13,5	17	5,6	36,2%	8,8	14,3	5,6	39,2%
	N	127				14			
TXA + LIA	M	10,7	13,8	3,1	22,7%	8,1	12,4	4,3	34,6%
	SD	1,2	1,2	0,9	6,4%	0,7	1,2	0,9	5,4%
	MD	10,5	13,7	3,2	23,4%	8,3	12,7	4,6	36,5%
	MIN	8,2	10,5	0,9	7,4%	5,9	9,9	2,5	23,9%
	MAX	14,7	17,8	6,3	41,7%	8,8	14,1	5,8	41,7%
	N	207				18			
Overall	M	10,6	14	3,4	24,4%	8,2	13	4,8	36,6%
	SD	1,2	1,2	1	6,4%	0,5	1,1	0,9	4,9%
	MD	10,4	13,9	3,4	24,8%	8,4	13,1	4,8	36,6%
	MIN	8,2	10,5	0,4	3,4%	5,9	9,9	2	19,4%
	MAX	15,2	19	6,5	41,7%	8,9	15,4	7,3	49,3%
	N	458				72			

**Fig. 3:**
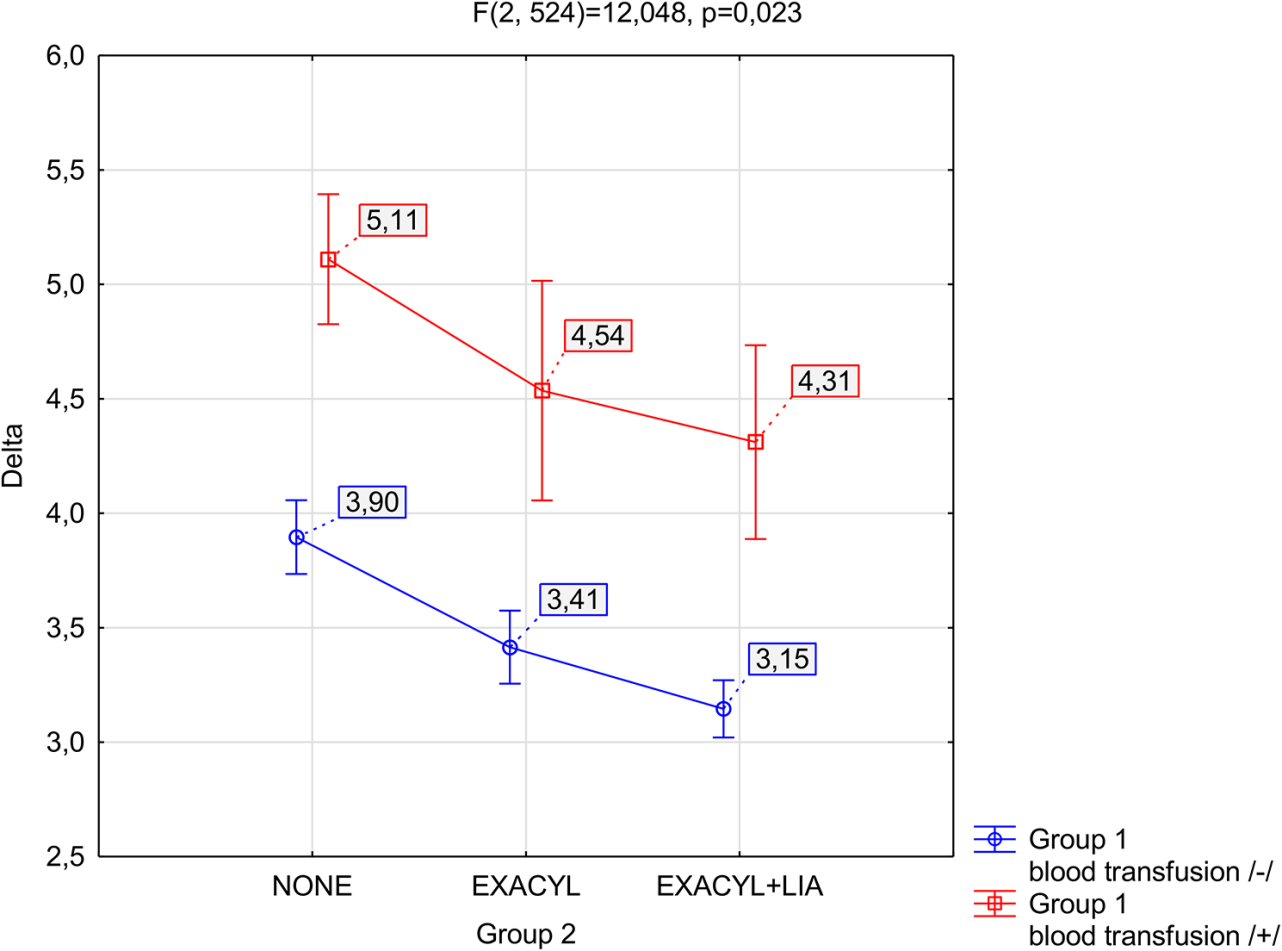
Mean plots with 95% confidence intervals (CIs) for the mean Hb decrease are presented separately for patients requiring (blue) and not requiring (orange) blood transfusion

The factorial ANOVA test and Tukey’s post hoc test for unequal samples were used to verify whether there were apparent differences between groups (Tables 3 and 4; supplementary material). Figure 3 shows the statistical data for the mean decrease in Hb. The 95% confidence interval for the mean indicates a range of results that will favour 95% of the tested people from a given group.

**Fig. 4:**
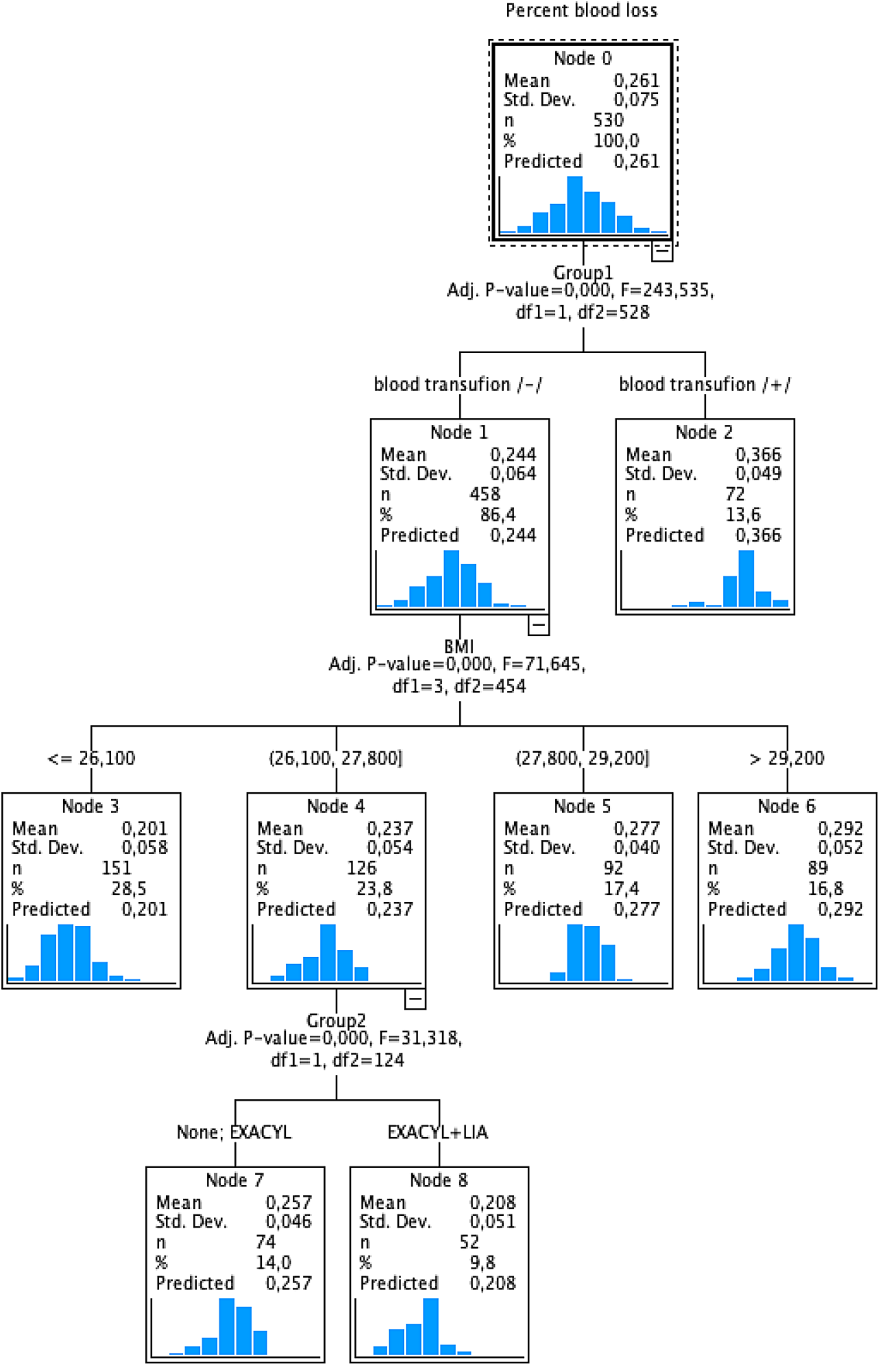
CHAID prediction tree model. A model considering variables, i.e., a group of patients who received and did not receive blood, divided into subgroups: control group, TXA group, TXA + LIA group, BMI, age, and gender

Sex and age were not significantly related to perioperative blood loss in any of the groups. However, BMI is strongly related to blood loss. It can be concluded that the lower the BMI, the lower the blood loss after TXA administration (*r* = 0.737; *p* < 0.01). Blood loss was consistently lower in the TXA + LIA group (*r* = 0.805; *p* < 0.01). Figure 4 shows a predictive model of the relationship between blood loss and BMI. The risk of blood loss in our study increased with increasing BMI by 20.1% for BMI < = 26.1, 23.7% for BMI 26.1–27.8, 27.7% for BMI 27.8–29.2, and 29.2% for BMI > = 29.2.

## Discussion

Several studies have proven that TXA administration in the perioperative setting is a safe and effective method for reducing blood loss [21, 22]. Numerous randomized controlled trials have confirmed the effectiveness of a dose of 1.0 g TXA, calculated according to a body weight cut-off of 20 mg/kg [23]. One dose of TXA should be administered, and two or more doses are typically administered. Iwai et al. showed that 2 doses of intravenous TXA during the perioperative period resulted in a greater reduction in blood loss than a single dose [24]. Furthermore, many studies have revealed that topical tranexamic acid has comparable efficacy in TKA and does not induce a prothrombotic effect [11, 25–27]. The effectiveness of combining intravenous and topical TXA can be found in the literature. This protocol results in a lower average blood loss volume and a lower decrease in haemoglobin levels than a single intravenous administration [28]. Marra F et al., in a prospective study, compared intravenous, intraarticular, and a combination of the two methods of administering TXA and concluded that no protocol seems superior to the others in terms of blood loss and decrease in haemoglobin level [29].

On the other hand, current evidence indicates a dose-dependent toxic effect of TXA on cartilage, tendon, and synovial tissue. Concentrations of 20 mg/ml or less are expected to be safe. In light of these studies, caution with intraarticular administration of TXA is suggested [30]. However, there are still concerns about potential adverse events; the decision on the utilization of TXA therapy should always depend on the balance between the efficacy and safety of the therapy.

Venous thromboembolic disease (VTED) is a known complication of TKA. Although patients undergoing knee replacement routinely receive anticoagulant prophylaxis, VTED can still occur, accompanied by deep venous thrombosis (DVT) or pulmonary embolism (PE). The prevalence of VTED in TKA patients is estimated to be 0.8 to 1.8% [31, 32]. In our study, we administered two doses of 1.0 g of TXA 30 min before the skin was incised and 3 h later. Like other authors, we did not notice an increased risk of VTED in our study group. We documented two cases of DVT and no cases of PE among 530 patients (0.4%). One patient with deep venous thrombosis was in the control group (66-year-old woman with a BMI of 29.7), and one was in the TXA + LIA group (75-year-old woman with a BMI of 29.8). Neither patient had additional risk factors for VTED, and both required blood transfusion due to a decrease in haemoglobin and systemic symptoms of anaemia. The low level of thrombotic complications in our study could be explained by the fact that patients with preexisting coagulopathy were excluded to ensure the homogeneity of the compared groups. In all the study groups, surgical procedures were performed without a tourniquet, and adequate haemostasis was achieved, thereby limiting intraoperative blood loss. In our centre, we decided to stop using a tourniquet because we wanted to avoid injuring the femoral muscles. Many studies have shown that TKA without a tourniquet can reduce early postoperative pain and improve rehabilitation without the risk of increasing side effects [33, 34].

One limitation of our study was the use of a drain in the control group. No drains were used in the TXA or TXA + LIA groups. Several studies have shown that using a drain in TKA increases blood loss [35, 36], while others have reported that using a drain does not influence perioperative blood loss [37–39]. Although there is no agreement on the use of a drain or its impact on perioperative total blood loss, we believe this may have caused a more significant difference between the study groups in our survey. Local infiltration analgesia is administered intraoperatively into the posterior capsule and soft tissues around the knee to reduce postoperative pain. There was heterogeneity in the studies on local analgesic drug combinations, infiltration sites, and volumes. Some LIA protocols rely on bupivacaine, while others rely on ropivacaine. The addition of steroids and nonsteroidal anti-inflammatory drugs has been questioned because of the potentially increased risk of periprosthetic infection and renal as well as gut toxicity [19, 40]. In close consultation with anaesthesiologists, we started routinely using LIA in TKA in January 2021. In contrast to most recommendations, we administered 100 ml of the LIA solution because high volumes of LIA solutions may increase the risk of prolonged wound drainage or leakage [19, 41]. Interestingly, our study showed no wound infection, delayed wound healing, or prolonged wound leakage in the TXA + LIA group. The patients in the LIA group had significantly lower scores on the visual analogue scale (VAS), especially in the first two postoperative days, than the other patients had. However, the detailed assessment was different from the purpose of this study. It is considered that although LIA is a recommended analgesic option, it cannot improve functional outcomes after TKA. Based on our results, the combination of TXA with LIA reduces perioperative blood loss. In our study, the average decrease in haemoglobin in the control group was 30.08%. The average decrease in haemoglobin was 25.17% in the TXA group (*p* < 0.001) and 23.67% (*p* < 0.001) in the TXA + LIA group. Moreover, reducing blood loss seems crucial for decreasing the rate of allogeneic blood transfusions because it could increase the risk of surgical site infection and periprosthetic joint infection [42]. For our patients, we noticed a statistically significant relationship (*p* < 0.01) between the decrease in the allogeneic blood transfusion rate, which was 24.4% in the control group, 9.9% in the TXA group, and 8% in the TXA + LIA group. Therefore, the combination of TXA and LIA might positively affect the final outcome of TKA. According to Başdelioğlu K, high BMI values adversely affect clinical and functional outcomes after TKA. Furthermore, obesity is one of the most critical risk factors for prosthesis infection and aseptic prosthesis loosening [43]. After analysing the results, we did not find any correlation between blood loss and sex or age. However, we found a strong relationship between blood loss and BMI. Our study revealed that the risk of blood loss increased with increasing BMI by 20.1% for BMI < = 26.1 kg/m2 and 29.2% for BMI > = 29.2 kg/m2.

The current study revealed no complications associated with the combination of LIA and TXA. We did not experience any intricacies resulting from administering LIA into the large vessel, nerve damage or severe local complications. Moreover, we did not find any adverse interactions associated with the combination of TXA and adrenaline; in particular, there was no increased risk of local prothrombotic state, which could result from the haemostatic effect of TXA and the vasoconstrictive effect of adrenaline. Based on our observations, combining LIA and TXA is safe and reduces pain and perioperative blood loss.

We believe that the combination of TXA and LIA allows for the reduction of perioperative blood loss through two separate mechanisms. On the one hand, a systemic effect through the intravenous use of tranexamic acid promotes hemostasis and reduces bleeding and blood loss by inhibiting activated plasmin from degrading fibrin clots [8, 9]. On the other hand, using epinephrine in LIA reduces perioperative (contraction of peripheral vessels through alpha-1 adrenoreceptors) and postoperative (platelet-stimulating hemostatic effect through alpha-2 adrenoreceptors) blood loss [44]. The key is to follow the LIA protocol to focus on the most richly vascularized knee structures. Intraoperative peri-incisional and pericapsular local injection allows for the most effective constriction of small vessels and reduces blood loss [45, 46].

In the original LIA technique, the local tumescent effect of large bolus infiltration into soft tissues was responsible for the hemostatic effect. We did not use high volumes in our study to avoid the increased risk of prolonged wound drainage or leakage. Moreover, in our opinion, the techniques employed in our survey, including thorough diathermy coagulation, tranexamic acid, and local infiltration, make it possible to avoid drainage, which additionally reduces the risk of postoperative allogenous blood transfusion, thus reducing the associated risks and costs [47].

The scheme of combining TXA with LIA that we have implemented in our study is consistent with Enhanced Recovery After Surgery (ERAS), which is a “fast-track,” perioperative, multi-disciplinary concept aimed at improving recovery time and reducing the length of hospital stay as with consensus for perioperative care in total hip and knee arthroplasty [48–50].

However, further analysis is needed to determine the impact of the combination of tranexamic acid and local infiltration analgesia on reducing perioperative blood loss and the rate of blood transfusions after total knee replacement.

### Study limitations

The main limitations of our study include its retrospective design. Another limitation of our study was the use of a drain in the control group, as a drain was not used in the TXA or TXA + LIA group, which could have influenced intraoperative blood loss in the control group. Another limitation is that the volume of intraoperative blood loss was not evaluated. Estimating the rate of blood transfusions and the lack of strict transfusion guidelines is another essential facet. According to local hospital regulations, the hemoglobin cut-off level for transfusion was 8.5 g/dl. However, the transfusion trigger was less than 9.0 g/dl in patients with anemia-related symptoms. Based on the guidelines, it is worth mentioning that transfusion triggers are not static and, especially in the perioperative period, may be dynamic and only partially dependent on hemoglobin levels, which may result in estimation bias. Limitations of the current study include the lack of comparisons between local and intravenous injections of tranexamic acid and the short postoperative follow-up period.

Further longitudinal studies comparing different methods of TXA administration with LIA are recommended.

## Conclusions

Compared to the separate administration of tranexamic acid, the combination of perioperative administration and local infiltration analgesia is a safe procedure that statistically significantly reduced blood loss in patients after total knee replacement. The combination of TXA and LIA reduces the rate of allogenic blood transfusion and shortens hospital stays.

### Electronic supplementary material

Below is the link to the electronic supplementary material.

Supplementary Material 1

## Data Availability

The data generated and analyzed in the current study are available from the corresponding author on reasonable request.

## Abbreviations

TKA: Total knee arthroplasty
THA: Total hip arthroplasty
TXA: Tranexamic acid
LIA: Local infiltration analgesia
NSAIDs: Non-steroidal anti-inflammatory drugs
STROBE: Strengthening the Reporting of Observational Studies in Epidemiology
LMWH: Low Molecular Weight Heparin
I&D: Irrigation & debridement procedure
VTED: Venous thromboembolic disease
DVT: Deep venous thrombosis
PE: Pulmonary embolism
