# Supplementary material for: Can local infiltration analgesia supplemented with tranexamic acid reduce blood loss during total knee arthroplasty?
Source: BMC Musculoskelet Disord. 2024 Apr 26;25:333. doi: 10.1186/s12891-024-07451-9 (PMC11046775; doi:10.1186/s12891-024-07451-9)
Supplement: Supplementary file 1 — Supplementary Material 1 [file 12891_2024_7451_MOESM1_ESM.docx]

**Supplementary materials.**

|  | SS | df | MS | F | p |
| --- | --- | --- | --- | --- | --- |
| **Intercept term.** | 3450,016 | 1 | 3450,016 | 4130,919 | p<0,01 |
| **Group 1 (blood transfusion /-/ and blood transfusion /+/)** | 70,930 | 1 | 70,930 | 84,929 | p<0,01 |
| **Gruup 2 (no blood protocol; TXA; TXA + LIA)** | 28,244 | 2 | 14,122 | 16,909 | p<0,01 |
| **Group 1* Group 2** | 10,083 | 2 | 6,041 | 12,048 | p=0,023 |
| **Error** | 437,629 | 524 | 0,835 |  |  |

***Table. 3. Factorial Anova statistics. SS – within-group variance; df- degrees of freedom; MS – between-group variance; F - Anova statistics; p-significance for F.***

|  |  |  | {1} M=3,90  **27,5%↓** | {2} M=3,42  **24,1%↓** | {3} M=3,15  **22,7%↓** | {4} M=5,11  **36,6%↓** | {5} M=4,54  **35,3%↓** | {6} M=4,31  **34,6%↓** |
| --- | --- | --- | --- | --- | --- | --- | --- | --- |
|  |  |  | p- Value | | | | | |
|  |  |  |  |  |  |  |  |  |
| **1** | **blood transfusion**  **/-/** | **CONTROL** |  | 0,028 | 0,001 | 0,000 | 0,005 | 0,038 |
| **2** | **blood transfusion**  **/-/** | **TXA** | 0,028 |  | 0,219 | 0,000 | 0,000 | 0,000 |
| **3** | **blood transfusion**  **/-/** | **TXA+LIA** | 0,001 | 0,219 |  | 0,000 | 0,000 | 0,000 |
| **4** | **blood transfusion /+/** | **CONTROL** | 0,000 | 0,000 | 0,000 |  | 0,009 | 0,000 |
| **5** | **blood transfusion /+/** | **TXA** | 0,005 | 0,000 | 0,000 | 0,009 |  | 0,041 |
| **6** | **blood transfusion /+/** | **TXA+LIA** | 0,038 | 0,000 | 0,000 | 0,000 | 0,041 |  |

***Table. 4. Duncan post-hoc test statistic***
